# Evaluating the influence of sleep quality and quantity on glycemic control in adults with type 1 diabetes

**DOI:** 10.3389/fendo.2023.998881

**Published:** 2023-02-22

**Authors:** Marta Botella-Serrano, Jose Manuel Velasco, Almudena Sánchez-Sánchez, Oscar Garnica, J. Ignacio Hidalgo

**Affiliations:** ^1^ Endocrinology and Nutrition Service, Hospital Universitario Príncipe de Asturias, Madrid, Spain; ^2^ Computer Architecture and Automation Department, Universidad Complutense de Madrid, Madrid, Spain; ^3^ Education Department, Universidad a Distancia de Madrid, Madrid, Spain

**Keywords:** sleep structure, glycemic control, clustering techniques, glucose behavior prediction, statistical analysis

## Abstract

**Background:**

Sleep quality disturbances are frequent in adults with type 1 diabetes. However, the possible influence of sleep problems on glycemic variability has yet to be studied in depth. This study aims to assess the influence of sleep quality on glycemic control.

**Materials and methods:**

An observational study of 25 adults with type 1 diabetes, with simultaneous recording, for 14 days, of continuous glucose monitoring (Abbott FreeStyle Libre system) and a sleep study by wrist actigraphy (Fitbit Ionic device). The study analyzes, using artificial intelligence techniques, the relationship between the quality and structure of sleep with time in normo-, hypo-, and hyperglycemia ranges and with glycemic variability. The patients were also studied as a group, comparing patients with good and poor sleep quality.

**Results:**

A total of 243 days/nights were analyzed, of which 77% (*n* = 189) were categorized as poor quality and 33% (*n* = 54) as good quality. Linear regression methods were used to find a correlation (*r* =0.8) between the variability of sleep efficiency and the variability of mean blood glucose. With clustering techniques, patients were grouped according to their sleep structure (characterizing this structure by the number of transitions between the different sleep phases). These clusters showed a relationship between time in range and sleep structure.

**Conclusions:**

This study suggests that poor sleep quality is associated with lower time in range and greater glycemic variability, so improving sleep quality in patients with type 1 diabetes could improve their glycemic control.

## 1 Introduction

Poor sleep quality and insufficient amount of sleep are common in the general population and people with type 1 diabetes mellitus (T1DM) (1, 2). A shorter duration of the deep sleep phase (3, 4), subjective quality of sleep, excessive daytime sleepiness (1, 2), and higher prevalence of obstructive sleep apnea (5) have been demonstrated in both adults and children with T1DM. The impact of these disturbances on glycemic control in patients with T1DM is an area of increasing interest. Previous studies suggest that sleep disturbances decrease insulin sensitivity, worse glycemic control, and increase glycemic variability (6). Recently, the American Diabetes Association recommended the study of sleep patterns as part of clin-ical evaluation of a patient with T1DM (7). The main objective of this study is to investigate by machine learning techniques the relationship among sleep structure, sleep quantity and quality, and glycemic control in patients with T1DM.

Griggs et al. (2020) (8) found in 38 patients that a higher glucose variability was associated within-person with more sleep disruptions or worse sleep. Our work extends theirs by grouping sleep patterns and analyzing the influence on glucose values during the day. Feupe et al. (2013) (9) studied the relation-ship between deep sleep duration and HbA1c level and concluded that they are inversely correlated.

Some previous studies used signal processing techniques to study the influence of physical exercise during the day on the glucose evolution during the following night (10). To find coherence between the patient’s circadian rhythms, they used the *cosinor* technique (a technique used in circadian physiology) and wavelets. Another similar study using the wavelet coherence analysis is Griggs et al. (2022) (11). Other studies (12–15) found significant relations between variability in sleep duration and poor glycemic control.

Our study complements these works by including the different sleep states during the night, grouping them into repetitive patterns, and studying their influence on different metrics of the following day. We apply clustering techniques and language processing techniques.

## 2 Materials and methods

The study was approved by the ethics committee of the *Príncipe de Asturias* Hospital of Alcalá de Henares, Madrid, Spain. The research was compliant with the Declaration of Helsinki guidelines. Written consent was obtained from each participant prior to engagement.

### 2.1 Inclusion/exclusion criteria

Eligible participants were adults between 18 and 65 years with T1DM with at least one year of duration, being on treatment with an insulin pump or multiple doses of subcutaneous insulin per day (MDI), having the availability of a mobile device capable of reading the sensors of the FreeStyle Libre system, and giving informed consent for inclusion in the study. Pre-screened subjects were excluded if they were diagnosed with a significant psychiatric disorder. Subjects in treatment with corticoids or patients that have required hospitalization or surgery on the last six months were excluded.

### 2.2 Data gathering and preprocessing

The main objective of this study is to analyze the impact of sleep disturbances on short-term gly-cemic control, glycemic variability, and the frequency of hypoglycemia in a group of patients with T1DM. For this purpose, flash continuous glucose monitoring (performed by Abbott FreeStyle Libre devices) and a sleep study using wrist actigraphy (Fitbit Ionic device on the non-dominant wrist) were carried out simultaneously for 14 days in a group of patients. The CGM data includes interstitial blood glucose levels recorded during the entire time the patient wore the sensor, not only during sleep. Fitbit ionic devices incorporate a light sensor (photoplethysmography, PPG) and an accelerometer to identify sleep stages. From (16), “Fitbit uses proprietary sleep-staging machine learning algorithms applied to mo-tion, heart rate variability, and respiratory rate, with the last two calculated from heartbeat data sensed by PPG”. Twenty-five patients were included, although data from three patients had to be discarded, with a total of 243 nights/days recorded. The study analyzes interindividual and intraindividual differences in glycemic control concerning nights with worse or better quality/quantity of sleep.

Three visits were programmed to complete the collaboration of the participants. The study was explained to the participants in a first visit (*Pre-screening Visit*), and all patients signed an informed consent form. Participants were also committed to continuing with their usual treatment. In addition, sociodemographic variables, anthropometric data, and clinical data were collected from medical records. In a second visit, *Visit 1*, participants were given a wristband with wristwatch actigraphy (Fitbit Ionic device), and a FreeStyle Libre sensor (first generation, no alarms) was placed for continuous glucose monitoring for 14 days. The sensor was connected to the Abbot Libre View platform. Patients self-completed the *Pittsburgh Sleep Quality Index (PSQI)* questionnaire to assess habitual self-perceived sleep quality (1). Visit 1 took place between 1 and 30 days after the pre-screening visit. Finally, during *Visit 2*, the glucose sensor and wrist actigraphy were removed. *Visit 2* was programmed to be held 15 days after *Visit 1*. During this period, participants could contact the study’s technical staff to solve any technical concerns.

PSQI examines seven components: sleep quality, latency, habitual sleep efficiency, sleep duration, sleep disturbances, use of sleep medication, and daytime dysfunction. With 19 questions, participants rate the components on a scale of 0 to 3, ranging from 0 to 21, with higher scores indicating worse sleep quality (>5 reveal poor sleepers).

Recording of blood glucose data was performed through the Abbott Libre View application. Time-in-range is estimated directly by the FreeStyle Libre systems. In addition, we calculated it from the microdata generated by the meter using the Rosendaal method (17), which assumes a linear progression between two glucose values and calculates the specific value for each minute (linear interpolation). The same method was used to calculate time in hypo and hyperglycemia. The recording of the wrist activity was also performed automatically and digitized by the Fitbit mobile application. Microdata is not available directly from Fitbit, so we adapted the API (Application Program Interface) for recovering detailed information (1).

Glucose sensors were placed on the arm, and the Fitbit device was worn on the non-dominant wrist. The Fitbit and glucose data were synchronized at the closest multiple of 5 minutes. Once synchronized, sleep times spent in each stage were added to resynchronize with the 15 minutes used by FreeStyle Libre data. Days with gaps higher than one hour and a half in glucose were discarded. Fitbit data presented some outliers in sleeping times, mixing nap and night sleeping times for some days. Those days were eliminated manually. Heart rate, steps, and burned calories were collected and synchronized for future studies. Limitations of Fitbit Ionic devices are discussed in section 5

The glucose monitoring variables analyzed in this study are: time-in-range 70-180 mg/dl (TR) in percentage, mean blood glucose (mg/dl) (Mean_glucose), standard deviation (SD), coefficient of vari-ation (CV), percentage of time spent in level 1 hypoglycemia (55-70 mg/dl) and level 2 hypoglycemia (*<* 55 mg/dl) (T Hypo), time in hyperglycemia level 1 (180-250 mg/dl) and level 2 (*>* 250 mg/dl) (T Hyper), number of hypoglycemia/hyperglycemia episodes with at least 15 min of duration, Mean Amplitude of Glycemic Excursions (MAGE) and Mean Daily Glucose Differences (MDGD).

### 2.3 Methodology

**Figure 1:**
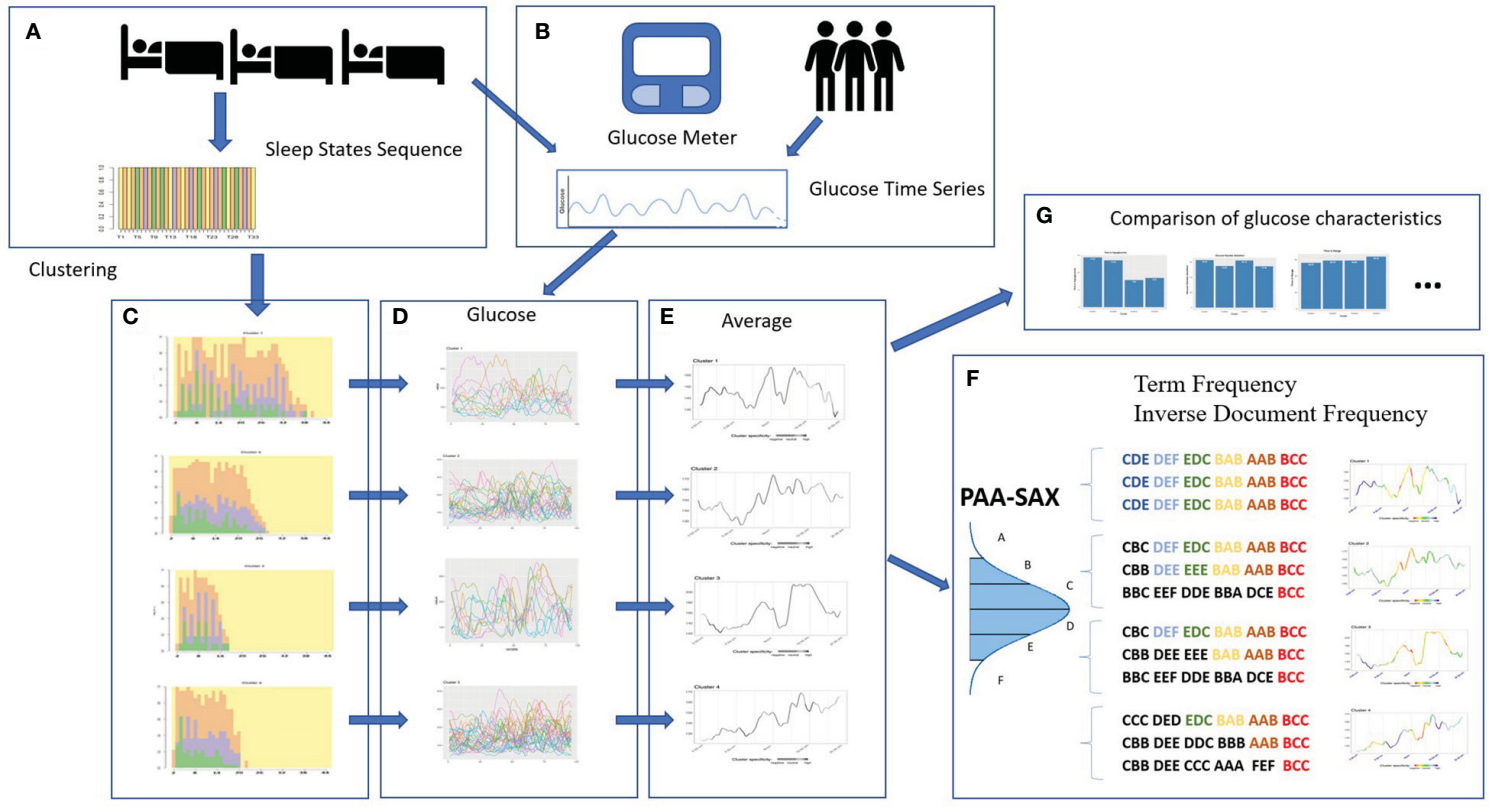
Sequence of steps for the development of our study: recording of sleep states and glucose levels **(A, B)**, clustering **(C)**, characterizing cluster glucose behavior **(D, E)**, finding specific glucose patterns **(F)** and comparison of glucose characteristics **(G)**. ^1^ https://python-fitbit.readthedocs.io/en/latest/.

**Figure 1** shows the workflow we have used in this study. First, we recorded the 24-hour time series of blood glucose levels (box B) and the sleep state sequences during the corresponding nights of the par-ticipants in the study (box A). After performing the clustering (subsection 2.4) according to the structure of sleep states (box C), we consequently grouped the daily time series of glucose levels corresponding to the nights of each cluster (box D). Then, the glucose time series were averaged by cluster (box E), and we obtained the dynamics of the glucose level that characterize each cluster. As a final phase, the behavior of the clusters is studied in two different ways: on the one hand, a language processing tech-nique is applied to find similarities and dissimilarities (subsection 2.5) in the glucose time series (box F) and, on the other hand, a statistical analysis (subsection 2.6) is performed to compare the glycemic characteristics between clusters (box G).

### 2.4 Analysis of the sequence of sleep states

Throughout the night, the person transits between different sleep states (wake, light, rem, deep), forming a time series of states or categories (18). **Figure 1** (box A) presents this time series as a sequence of colors displaying the sleep states. Each sleep state is represented by a color.

In this study, we want to determine whether there are patterns in the sleep time series during nights that correlate to patterns of blood glucose level evolution of the following day. To search for sleep patterns, we tried different time series clustering techniques (19). Clustering is a group of machine learning techniques that identify *clusters* in the data. A cluster is a group or subset of elements of a population.

In this work, we applied clustering to group the nights of the participants based on the sequence of sleep states, i.e., each cluster includes those temporal sequences that are most similar to each other (18). Subsequently, we analyzed the evolution of glucose during the following day for each cluster. Finally, we analyzed specific and expected behaviors in the diurnal evolution of glucose values for the different clusters. This last step is explained in more detail in subsection 2.5.

**Figure 1** (box C) illustrates the result of applying clustering to the sleep data in four clusters, i.e., four sleep behavior patterns of the study participants.

### 2.5 Similarity among glucose time series

**Figure 1** (box E) shows each cluster’s average glucose time series. To identify specific behaviors of each cluster, we applied techniques commonly used in language processing (20, 21). To do this, we trans-form a time series of numerical glucose values into a sequence of symbols. These symbols are obtained after the time series is normalized and reduced by obtaining the average of a number *n* of glucose values (in this work, *n* =4) (Piecewise Aggregate Approximation, PAA) (22). A symbol is assigned to each aver-aged point within a dictionary based on the statistical distribution (Symbolic Aggregate approXimation, SAX) (20, 21). Finally, the symbols are grouped into words of a specific size (12 in this work).

Next, we identify behaviors specific to each cluster and those familiar to all clusters. Based on the number of occurrences of each “word” in all the time series of each cluster, we obtain the weight vectors associated with each word [Term Frequency -Inverse Document Frequency (23)]. Thanks to the weight vectors, we calculate the cosine similarity (24) and use this value to know if a word is specific to a cluster. In the average time series, we show those cluster-specific segments in cool colors (dark and light blue) and warm colors (red and orange) the similar segments across all clusters. The concept is that words with a very high frequency of occurrence in one cluster and a shallow frequency in the others appear in blue, whether the word appears a lot in all the clusters appears in orange or red. If the occurrence in the other clusters is medium, the color is green or yellow. Hence, we can identify the dynamics of glucose that characterizes a cluster. In **Figure 1** (box F), we present a summary of the process for words of size

### 2.6 Statistical methods

In order to find out the possible relationship between the glucose levels and the quality of sleep, several cluster analyses and correlational studies were performed using the R language and related libraries (18, 25). These types of models, together with language processing techniques taken from the field of artificial intelligence, make it possible to determine possible patterns between the different variables selected and the nocturnal sleep patterns (26).

On the one hand, the K-means algorithm is used in the various cluster analyses among available data (27): first, considering the variables associated with sleep alone, then the variables associated with glucose levels, and finally, the set of all variables. On the other hand, the correlation analysis takes as a reference Pearson’s correlation coefficient. These values have been obtained after processing these glucose records with the R Package *gluvarpro* (28).

To compare the glycemic characteristics of clusters, we used Welch’s F-test (29) using the package from Dag et al. (2018) (30). We performed pairwise tests using Bonferroni’s correction (31, 32) for the p-values to calculate pairwise differences for each variable between the scores of each cluster (using the same package as before). In addition, we used Shannon’s entropy (33) for analyzing the results of the clustering. Shannon’s entropy provides an idea of how ordered sleep was. Higher values of entropy indicate higher levels of disorder.

## 3 Results

### 3.1 Participant characteristics

Twenty-five subjects participated in this study, of whom fourteen were female and eleven were male. **Table 1** shows the characteristics of the participants identified by a random ID and including gender (M=Male; F=Female), age, BMI, HbA1c, diabetes treatment (MDI: Multiples doses of insulin; CSII: Continuous subcutaneous insulin infusion), and years of evolution of T1DM.

**Table 1:** Characteristics of the participants: ID, Gender (M, Male; F, Female; Sex assigned at birth coincides with gender identity for all participants), Age, BMI, HbA1c, Treatment (MDI, Multiples doses of insulin; ISCI, Infusion Subcutaneal continuous of insulin); Years of evolution of T1DM.

ID	Gender	Age	BMI	HbA1c	Treatment	Years T1DM
**HUPA001**	F	Q4	Q2	Q4	ISCI	Q3
**HUPA002**	M	Q4	Q2	Q2	ISCI	Q4
**HUPA003**	F	Q3	Q1	Q2	ISCI	Q2
**HUPA004**	M	Q2	Q4	Q3	ISCI	Q1
**HUPA005**	F	Q1	Q2	Q1	ISCI	Q4
**HUPA006**	M	Q1	Q3	Q3	ISCI	Q2
**HUPA007**	M	Q2	Q4	Q1	ISCI	Q1
**HUPA008**	F	Q1	Q4	Q4	ISCI	Q1
**HUPA009**	F	Q3	Q2	Q3	ISCI	Q4
**HUPA010**	F	Q3	Q1	Q1	ISCI	Q2
**HUPA011**	F	Q2	Q2	Q3	ISCI	Q4
**HUPA014**	F	Q4	Q3	Q4	MDI	Q2
**HUPA015**	F	Q3	Q1	Q1	MDI	Q1
**HUPA016**	F	Q2	Q3	Q1	ISCI	Q3
**HUPA017**	F	Q1	Q1	Q4	MDI	Q3
**HUPA018**	F	Q2	Q1	Q2	ISCI	Q4
**HUPA019**	M	Q1	Q3	Q2	MDI	Q1
**HUPA020**	M	Q3	Q3	Q4	MDI	Q2
**HUPA021**	F	Q4	Q3	Q3	MDI	Q1
**HUPA022**	M	Q4	Q2	Q1	ISCI	Q2
**HUPA023**	M	Q1	Q1	Q3	MDI	Q1
**HUPA024**	M	Q3	Q4	Q4	MDI	Q4
**HUPA025**	M	Q2	Q4	Q1	ISCI	Q3
**HUPA026**	F	Q4	Q4	Q2	MDI	Q3
**HUPA027**	M	Q1	Q1	Q2	MDI	Q3
**Average**	F:14/25	38.3	24.4	7.4	ISCI:15/25	18.1

The mean age is 38.3 years, with an age range of 18-60.8 years, while the first and third quartiles are 26.4 and 47.9 years. The mean duration of diabetes is 18.1 years with a range of 0.8-39.5 years, and the first/third quartiles are 11.2/24.2 years. HbA1c mean is 7.4% (range 6-9.7%) and 1st/3rd quartiles are 7/7.8%. Body Mass Index (BMI) mean is 24.4 (range 18.5-32.2) and 1st/3rd quartiles are 22.3/26.3. Fifteen patients were on continuous insulin pump therapy, and ten were on multiple daily insulin doses. The CGM shows a mean blood glucose of 155 mg/dl, high glycemic variability (CV 36), and a time in hypo and hyperglycemia above target.

### 3.2 Pittsburgh questionnaire results

**Table 2** shows the results of the Pittsburgh questionnaire. The results of the PSQI give a poor overall sleep quality in 12/23 patients, being these results concordant with the objective assessment of the actigraphy. Sleep disturbances contribute most to the high PSQI score (sudden nocturnal awakenings or other reasons like heat, cold, pain, nightmares, snoring, coughing, or the need to urinate). This result is also concordant with the actigraphy results, where the mean duration of objective nocturnal awakenings (WASO) is 52 minutes. Nine patients report significant daytime dysfunction (score >1) regarding drowsiness or poor mood for daily activities. One of the participants was discarded because he/she worked in shifts.

**Table 2:** Results of Pitsburg questionnaire.

ID	Quality	Latency	Duration	Efficiency	Disturbances	Medication	Di dysfunction	Global	Subjective hours sleep
HUPA001	3	3	1	0	3	0	3	13	6
HUPA002	0	1	1	0	1	1	2	6	7
HUPA003	3	2	3	0	2	0	2	12	5
HUPA004	0	1	0	0	1	3	2	7	8
HUPA005	1	1	1	0	2	0	0	5	7
HUPA006	0	1	1	1	1	0	2	6	6.3
HUPA007	0	1	1	0	1	1	1	5	7
HUPA008	0	1	2	0	1	1	1	6	5
HUPA009	0	1	1	0	1	0	2	5	6.3
HUPA010	2	3	3	3	2	0	3	16	4
HUPA011	1	1	0	0	1	0	1	4	7.3
HUPA014	2	2	1	3	2	0	0	10	7
HUPA015	1	0	0	0	1	0	1	4	6.3
HUPA016	1	1	0	0	1	0	0	3	7.3
HUPA017	2	1	3	3	2	0	3	15	4.3
HUPA018	2	1	1	1	1	0	1	8	5
HUPA019	1	1	0	0	1	0	0	3	10
HUPA020	0	1	0	0	1	0	1	4	6.3
HUPA021	3	3	1	1	2	0	0	12	6
HUPA022	0	NV	0	NV	NV	0	1	NV	6
HUPA023	0	2	0	0	1	0	0	3	8.3
HUPA024	0	3	2	1	1	0	0	7	5
HUPA025	0	0	1	2	1	1	0	5	6
HUPA026	1	1	0	0	2	3	1	10	8
HUPA027	1	1	0	0	1	0	0	3	7.5
Average	0.91	3.8	1.38	1,19	0.57	1.33	0.32	1,29	7.09

### 3.3 Sleep and glycemic control characteristics

**Table 3** presents the values of the sleep monitoring variables for the participants of the study. Al-though most participants have a sleep efficiency higher than 90%, there are also 3 cases with a value close to 45% and two others with low-efficiency values (58% and 68%). Sleep data from some parti-cipants, such as HUPA007 or HUPA008, were discarded due to inconsistency in the reported data.

**Table 3:** Sleep States: Average duration with standard deviation (in minutes).

Participant ID	Efficiency %	Asleep (min)	Light (min)	Deep (min)	REM (min)	Awake (min)	Bed (min)
HUPA001	94±2	366.08±114.66	218.08±49.39	53.38±30.51	94.62±43.92	42.38±13.93	419±128
HUPA002	97±2	390.73±74.93	216.27±69.91	84.36±19.82	90.09±23.34	33.36±14.53	424±87
HUPA003	93±3	335.75±98.39	228.50±83.39	46.75±16.27	60.50±20.89	45.25±22.80	350±98
HUPA004	96±2	333.00±43.97	207.00±29.39	67.30±16.93	58.70±30.56	40.10±7.75	376±44
HUPA005	58±14	350.88±54.08	211.38±25.62	69.12±24.19	70.38±23.13	50.38±10.68	401±58
HUPA006	47±33	384.17±49.01	233.67±27.35	66.17±13.35	84.33±20.3	68.33±22.44	467±57
HUPA007	94±2	325.46±38.89	204.38±38.82	54.62±15.08	66.46±23.48	37.92±7.76	100±74
HUPA011	91±3	386.46±41.92	255.23±30.75	56.15±10.67	75.08±26.24	55.92±11.84	442±43
HUPA014	93±2	481.92±98.54	293.67±60.12	78.58±23.78	109.67±44.29	86.75±35.94	569±127
HUPA015	94±1	417.46±90.13	250.08±74.14	72.92±17.64	94.46±30.54	54.23±21.19	476±106
HUPA016	97±2	416.69±40.41	255.85±36.96	62.54±14.31	98.31±20.66	53.08±16.17	474±48
HUPA017	90±3	404.85±54.43	245.62±55.28	61.85±15.73	97.38±24.81	66.00±19.94	471±66
HUPA018	47±5	408.82±37.31	205.91±33.48	76.27±20.58	126.64±35.54	51.64±9.64	403±24
HUPA019	93±2	363.50±37.44	253.50±23.93	72.00±30.01	38.00±24.98	60.33±19.25	424±52
HUPA020	46±9	319.90±80.52	202.50±67.91	57.00±18.37	60.40±19.73	48.70±19.97	369±94
HUPA021	69±2	387.00±20.65	240.75±26.79	61.00±18.23	85.25±27.62	56.88±13.29	444±28
HUPA022	94±3	303.86±81.22	204.07±56.36	38.71±17.51	61.07±30.62	41.86±13.4	346±92
HUPA023	96±1	421.00±72.27	275.90±44.03	73.40±15.64	71.70±28.29	55.10±9.35	509±43
HUPA024	92±4	298.00±68.25	213.00±55.61	34.33±9.00	50.67±16.63	56.17±16.34	170±138
HUPA025	92±3	383.50±51.79	232.00±42.67	72.75±13.50	78.75±20.05	55.75±13.14	80±103
HUPA026	61±6	437.06±36.85	241.50±33.85	82.81±17.17	112.75±26.56	55.88±12.10	325±219
HUPA027	93±2	345.31±48.17	189.23±29.65	77.77±13.74	78.31±19.97	45.85±11.12	449±73
Average	83±5	375.52±60.63	230.82±45.25	64.54±17.82	80.16±26.46	52.81±15.57	391±119

Following the consensus on sleep quality assessment of the National Sleep Foundation (34), three vari-ables were used for evaluating the sleep quality: the number of awakenings during the night, WASO or Wake After Sleep Onset and the sleep efficiency (as the ration of total sleep time to time in bed (35).

**Table 4** shows the sleep characteristics of the participant. Sleep quality was categorized as poor if at least two of three mentioned criteria were met, i.e., sleep efficiency *<* 85% or Wake After Sleep Onset (WASO) *>* 40 min or a number of awakenings *>* 4. The sleep characteristics of the participants show large inter-individual differences, and only 48% of the patients have a good overall sleep quality, although the mean sleep duration is not low (mean of 7.15 hours). Of the 243 nights analyzed, 77% (*n* = 189) were of poor sleep quality and 33% (*n* = 54) of good quality. It should be noted that eight patients had no night with good sleep quality. The factor that most determined poor sleep quality was the duration of nighttime awakenings, with a mean of 52.81 minutes.

**Table 4:** Participants characteristics Sleep quality was categorized as ’poor’ if at least two of three criteria were met, i.e., sleep efficiency *<* 85% or WASO *>* 40 min or number of awakenings *>* 4, based on the National Sleep Foundation’s consensus recommendations for sleep quality assessment.

Participant	Total	Sleep Quality		Sleep Time	Sleep Efficiency	WASO	Awakenings
*ID*	*Nights*	*Good*	*Poor*	*Avg (hours)*	*Avg (%)*	*Avg (min)*	*Avg per night*
**HUPA001P**	13	3	10	6.81±2.11	94.08±2.14	42.38±13.93	5.00±2.24
**HUPA002P**	11	8	3	7.07±1.45	97.45±1.63	33.36±14.53	2.73±1.90
**HUPA003P**	12	7	5	6.35±1.99	93.08±2.81	45.25±22.8	3.25±1.76
**HUPA004P**	10	6	4	6.22±0.75	95.80±1.81	40.10±7.75	3.20±1.40
**HUPA005P**	8	0	8	6.69±0.97	58.50±14.41	50.38±10.68	3.50±1.51
**HUPA006P**	6	0	6	7.54±0.98	47.67±33.68	68.33±22.44	6.33±3.33
**HUPA007P**	13	8	5	6.06±0.68	94.08±2.10	37.92±7.76	2.69±1.49
**HUPA011P**	13	1	12	7.37±0.72	90.92±3.43	55.92±11.84	4.23±1.83
**HUPA014P**	12	1	11	9.48±2.12	92.67±2.39	86.75±35.94	5.08±2.50
**HUPA015P**	13	3	10	7.94±1.76	93.69±1.55	54.23±21.19	3.69±1.49
**HUPA016P**	13	2	11	7.91±0.80	96.54±2.22	53.08±16.17	0.15±0.38
*ID*	*Nights*	*Good*	*Poor*	*Avg (hours)*	*Avg (%)*	*Avg (min)*	*Avg per night*
**HUPA017P**	13	1	12	7.85±1.11	90.31±3.35	66.00±19.94	4.85±1.68
**HUPA018P**	11	0	11	7.67±0.69	46.91±5.49	51.64±9.64	4.09±1.14
**HUPA019P**	6	0	6	7.06±0.87	93.00±1.67	60.33±19.25	6.17±5.00
**HUPA020P**	10	0	10	6.14±1.57	46.50±9.16	48.7±19.97	3.70±1.25
**HUPA021P**	8	0	8	7.40±0.47	69.38±2.20	56.88±13.29	4.62±1.85
**HUPA022P**	14	8	6	5.76±1.54	93.93±2.97	41.86±13.40	3.50±1.56
**HUPA023P**	10	0	10	7.93±1.33	95.50±1.18	55.10±9.35	4.80±1.40
**HUPA024P**	6	1	5	5.90±1.38	92.00±4.47	56.17±16.34	1.17±2.86
**HUPA025P**	12	1	11	7.32±1.01	92.08±3.00	55.75±13.14	4.17±2.52
**HUPA026P**	16	0	16	8.22±0.71	60.75±5.86	55.88±12.10	5.31±1.82
**HUPA027P**	13	4	9	6.52±0.81	93.38±2.33	45.85±11.12	3.23±1.54
**Overall**	243	54	189	7.15±1.17	83.1±4.99	52.81±15.57	3.88±1.93

**Table 5** shows the percentages of time spent in sleep phases of Light, REM and Deep. Light phases percentage ranges from 45% to 60.8% with an average of 54.14%. The average time spent in the Deep phase is 15.08%, with a maximum of 20.19% and a minimum of 11.72%. Finally, participants spent an average of 18.49% of the time in bed in the REM phase, ranging from 8.62% to 27.24%.

**Table 5:** Sleep States: Ratio over Total Sleeping Time.

Participant ID	Light%	Deep%	REM%
HUPA0001P	54.85	12.49	22.23
HUPA0002P	50.23	20.19	21.88
HUPA0003P	59.51	12.68	16.36
HUPA0004P	55.71	17.98	15.47
HUPA0005P	53.25	16.93	17.19
HUPA0006P	51.74	14.55	18.74
HUPA0007P	56.18	15.01	18.33
HUPA0011P	57.72	12.72	16.85
HUPA0014P	52.26	14.00	18.88
HUPA0015P	52.07	15.76	20.00
HUPA0016P	53.85	13.26	20.88
HUPA0017P	51.83	13.51	20.76
HUPA0018P	45.00	16.56	27.24
HUPA0019P	60.80	16.55	8.62
HUPA0020P	54.46	15.25	17.30
HUPA0021P	54.44	13.65	19.17
HUPA0022P	59.13	11.72	17.05
HUPA0023P	58.11	15.43	14.81
HUPA0024P	59.81	10.19	14.20
HUPA0025P	52.66	16.63	18.07
HUPA0026P	49.04	16.83	22.83
HUPA0027P	48.32	19.87	19.94
Average	54.14	15.08	18.49

**Table 6** shows the overnight glycemic characteristics of the participants. Patients presented low values of time in range, with an average of 59.97 ± 14.74%, which is an indication of poor glycemic control. The high values of the average CV (36.45 ± 8.76) and standard deviation of the mean glucose (55.85 ± 14.) are also concordant with this appreciation.

**Table 6:** Overnight glycemic characteristics in individual participants.

Participant ID	Nights	Mean glucose mg/dl	SD	CV	TR	T Hyper %	T Hypo %
**HUPA001P**	13	181.71±32.27	67.12±10.97	37.27±5.07	54.21±13.77	43.54±14.74	2.25±3.13
**HUPA002P**	11	113.68±30.59	50.18±15.85	44.72±11.52	60.94±16.59	15.00±17.30	24.07±18.09
**HUPA003P**	12	139.88±22.85	55.38±16.57	38.98±5.56	69.66±12.53	22.69±14.68	7.65±7.24
**HUPA004P**	10	178.75±44.75	74.15±23.86	44.07±14.86	44.37±19.36	43.70±22.39	11.93±13.73
**HUPA005P**	8	151.06±23.74	43.05±14.68	29.29±11.12	69.09±16.96	27.21±18.19	3.70±4.45
**HUPA006P**	6	212.05±97.73	62.41±36.48	35.97±20.23	46.76±28.69	48.68±29.74	4.56±5.55
**HUPA007P**	13	173.64±29.53	73.14±14.31	42.53±8.47	46.28±17.35	45.07±17.61	8.65±8.49
**HUPA011P**	13	159.30±19.37	54.00±8.38	34.09±4.97	65.47±10.23	31.96±11.56	2.57±3.52
**HUPA014P**	12	186.47±19.12	68.55±22.54	36.68±11.00	44.96±10.21	50.83±11.34	4.20±3.77
**HUPA015P**	13	165.90±20.72	65.03±10.67	39.33±5.08	57.68±12.01	38.60±13.38	3.72±3.22
**HUPA016P**	13	157.32±45.81	67.24±20.44	43.51±12.62	51.16±18.54	36.10±22.75	12.74±10.33
**HUPA017P**	13	198.39±27.38	62.06±16.51	31.67±8.14	37.39±16.94	60.25±18.30	2.35±3.36
**HUPA018P**	11	144.12±35.97	62.64±17.93	43.60±5.62	49.73±11.83	31.77±18.56	18.50±14.91
**HUPA019P**	6	159.97±17.59	54.82±5.05	34.40±2.64	59.14±11.14	36.85±11.92	4.01±2.24
**HUPA020P**	10	193.99±29.73	72.11±18.31	37.23±7.74	44.92±14.04	51.29±15.31	3.78±3.88
**HUPA021P**	8	141.27±14.84	44.83±5.46	31.92±4.32	74.07±9.15	22.78±10.68	3.14±5.71
**HUPA022P**	14	111.12±23.51	31.58±7.55	29.00±6.96	79.57±11.25	5.01±8.62	15.42±13.42
**HUPA023P**	10	132.89±21.29	38.98±7.22	29.53±4.81	78.92±12.69	18.05±14.39	3.03±4.47
**HUPA024P**	6	157.51±31.58	56.53±18.80	37.25±14.74	57.63±9.82	35.17±15.25	7.19±10.08
**HUPA025P**	12	113.51±14.56	36.46±9.21	32.09±7.44	79.58±11.24	7.42±6.85	12.99±8.94
**HUPA026P**	16	133.64±24.60	57.68±20.78	42.86±11.98	60.70±21.91	22.25±17.37	17.05±13.76
**HUPA027P**	13	121.24±21.16	30.73±8.24	25.82±7.92	87.04±18.09	8.36±18.09	4.60±4.91
**Overall**	243	155.79±29.49	55.85±14.99	36.45±8.76	59.97±14.74	31.94±15.86	8.10±7.60

### 3.4 Association between sleep quality and blood glucose

**Figure 2:**
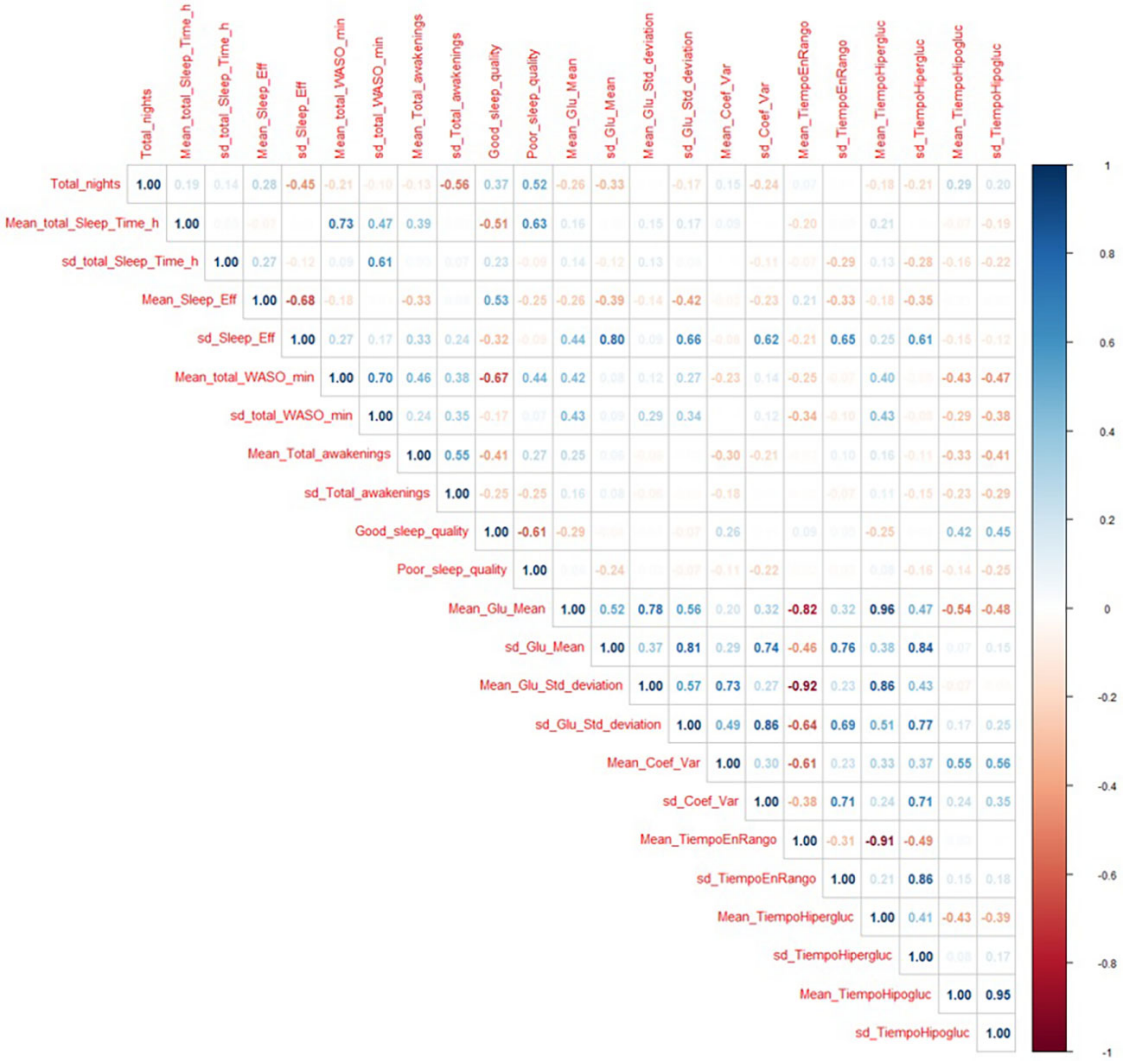
Correlation matrix for all the variables recorded in this study.

In **Figure 2**, the upper triangular view of the correlation matrix for different variables recorded in this study is displayed as a correlogram. Red/blue colors for showing negative/positive correlation, and low/high intensity indicating the absolute value of the correlation. In addition to the expected correlations, we can point out several facts. Both poor and good sleep qualities have no significant correlation with any other variable. A positive correlation of 0.8 was found between the standard deviation of sleep efficiency and the standard deviation of mean blood glucose. In addition, the standard deviation of sleep efficiency has a positive correlation (0.66/0.62/0.65/0.61) with the standard deviation of glucose, the coefficient of variation, the time in range, and the time in hyperglycemia. There is a positive correlation (0.55) between the coefficient of variation and the mean time spent in hypoglycemia.

As mentioned, we used the clustering techniques to find patterns in sleep behavior. We experi-mented with a different number of clusters, having found *k* =4 to be the best option, showing a clear relationship between sleep structure and glucose variables. With *k* =3, we have a cluster with very broad glucose patterns, whereas, with *k* =5, the relationship between sleep structure and the different glucose variables is confirmed with no additional information, remaining the main clusters the same. With *k* =4, the number of nights grouped in each cluster was: 33, 78, 41 and 91.

**Figure 3:**
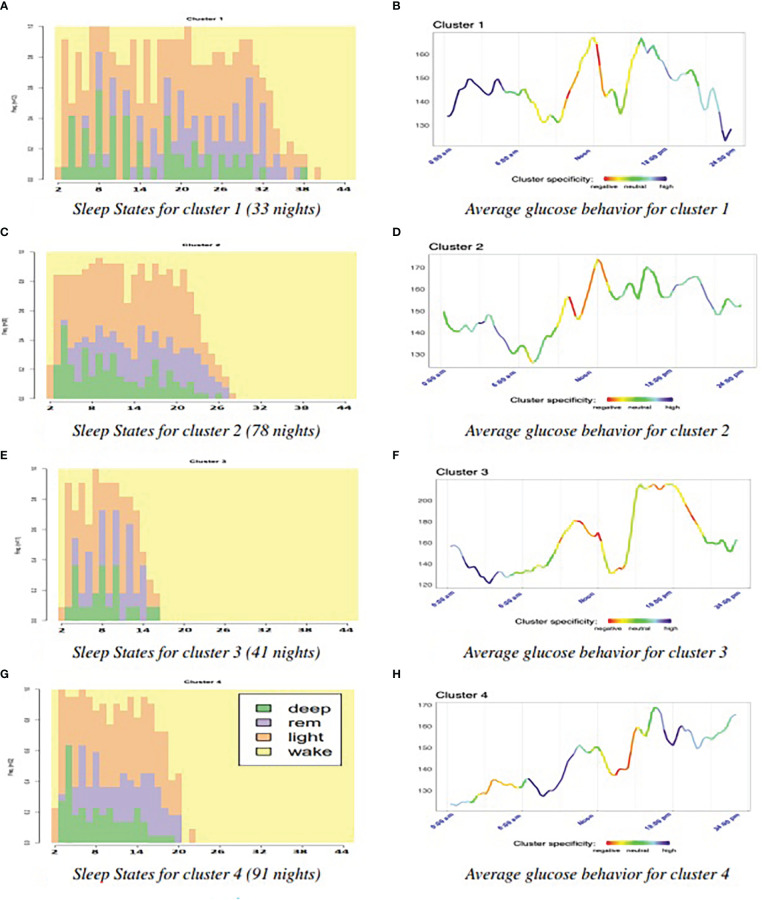
Analysis of specific patterns for each cluster. **(A)** Sleep States for cluster 1 (33 nights) **(B)** Average glucose behavior for cluster 1. **(C)** Sleep States for cluster 2 (78 nights) **(D)** Average glucose behavior for cluster 2. **(E)** Sleep States for cluster 3 (41 nights) **(F)** Average glucose behavior for cluster 3. **(G)** Sleep States for cluster 4 (91 nights) **(H)** Average glucose behavior for cluster 4.

In the left column of **Figure 3**, we can see the four sleep clusters, while the right column shows the average glucose dynamics in the days corresponding to each cluster. Remind that in the right column, the specificity of the glucose patterns is shown with intense blue color, while the patterns common to all clusters are shown in red. Because we take glucose level samples every 15 minutes, we have 96 samples per day. In the horizontal axis, we mark the main hourly correspondences. In order to correctly compare the sleep clusters, we show a total of 44 possible states per night (horizontal axis, on the left column).

**Figure 4:**
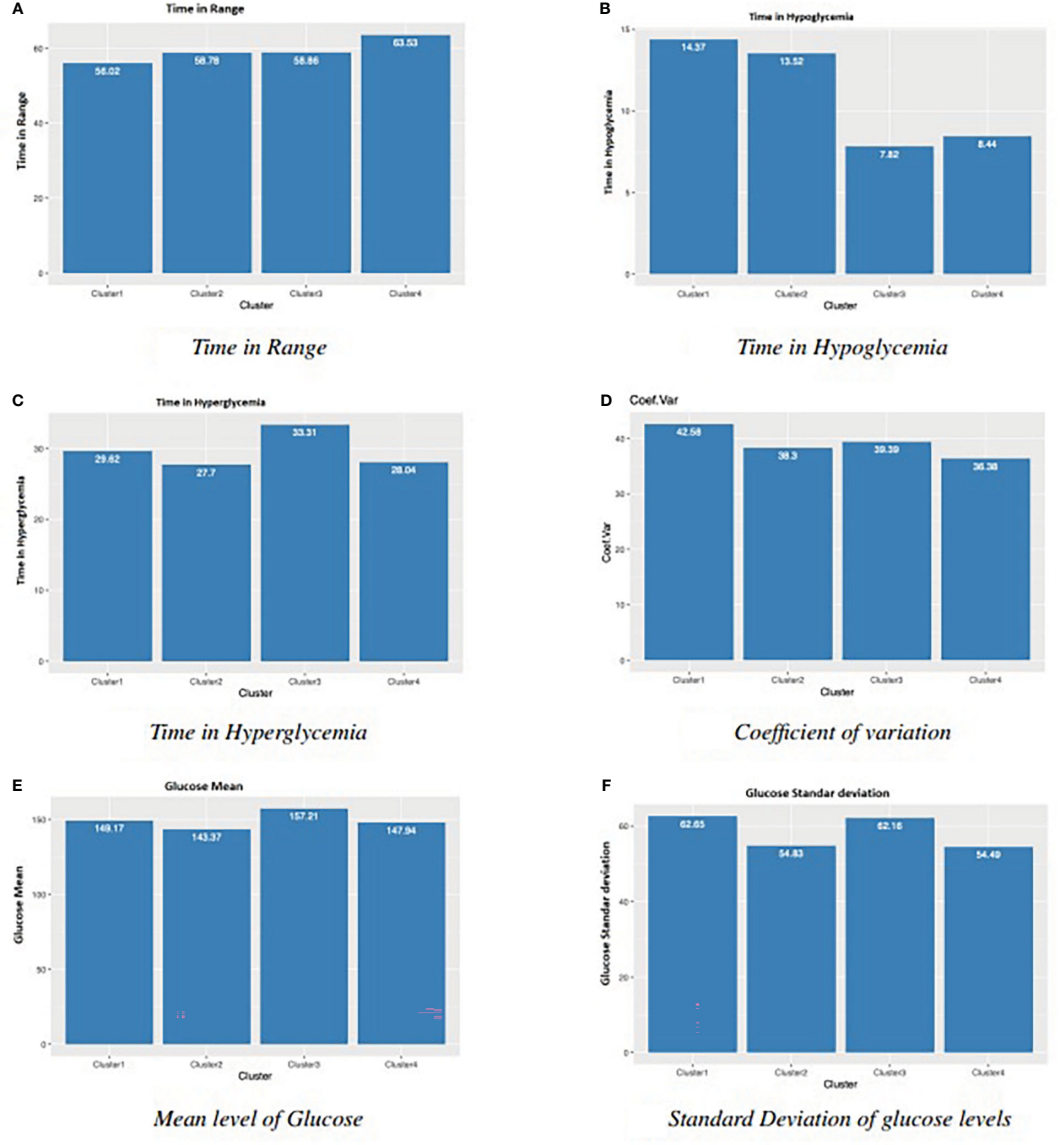
Main glucose related results for four clusters in **Figure 3**. **(A)** Time in Range **(B)** Time in Hypoglycemia. **(C)** Time in Hyperglycemia **(D)** Coefficient of variation. **(E)** Mean level of Glucose **(F)** Standard Deviation of glucose levels.

We calculated the Shannon’s entropy for the four clusters resulting in the following order (from highest entropy to lowest): 1,3, 2, and 4. **Figure 4** shows the results of the main glucose variables for each cluster. On the one hand, the four clusters have slightly different sample sizes. On the other hand, we cannot assume that the clusters will have the same variance. The result of Welch’s F-test indicates that we can reject the null hypothesis that each cluster has the same mean value.

After the statistical tests with Bonferroni’s correction, we obtain several observations. Regarding time in Range, there is statistical significance between Cluster 1 and Cluster 4. We found significant differences for Cluster 1 and Cluster 2, versus Cluster 3 and Cluster 4 in time in hypoglycemia, and for Cluster 3 versus Cluster 2 and Cluster 4 in time in hyperglycemia. Cluster 1 and Cluster 4 are also significantly different in terms of CV. For the mean of glucose values, despite Welch’s F-test, the pairwise tests found no significant differences. One explanation could be that the applied Bonferroni’s adjustment was too severe (36, 37). Another variable related to glucose variability, the standard deviation of glucose levels, presented significant differences for Cluster 1 and Cluster 3 versus Cluster 2 and Cluster 4. So, from these statistical results and the parallel observation of Figures (3 and 4 several conclusions arose. Clusters 1 and 2 (**Figures 3A, C**) have the longest sleep state sequences, the highest nocturnal glucose levels and a maximum peak around noon. Clusters 3 and 4 (**Figures 3E, G**), with the shortest sleep state sequences have the lowest nocturnal glucose levels and a maximum peak around sunset. In addition, these are the two clusters with the lowest time in hypoglycemia (**Figure 4B**). In the case of Cluster 3, this is mainly because this cluster has the longest time in hyperglycemia (**Figure 4C**), while Cluster 4 is the cluster with the longest time in range (**Figure 4A**). Cluster 1, with the highest Shannon’s entropy and the longest sequence of states (40), has as distinctive characteristic a very pronounced drop in glucose levels prior to the night and, at the same time, the most accentuated nocturnal rise (displayed in dark blue in **Figure 3B**). In addition, it is the cluster with the lowest time in range (**Figure 4A**), the highest time in hypoglycemia (**Figure 4B**), coefficient of variation (**Figure 4D**) and standard deviation (**Figure 4F**). Cluster 3, with the shortest sleep sequence (16 states), has the unique characteristic of a pronounced drop in glucose level during the night (**Figure 3F**). It is also the cluster with the highest time in hyperglycemia (**Figure 4C**) and, therefore, the highest mean level of glucose (**Figure 4E**). In **Figure 4F**, we can see that the lowest standard deviation corresponds to clusters 2 and 4. This could be related to the fact that these two clusters have the sleep state sequences with the lowest Shannon’s entropy. The shortest time in hyperglycemia and therefore lower mean glucose level (**Figures 4C, E**) corresponds to Cluster 2, which has a low level of Shannon’s entropy and a medium length of the sleep state sequence (**Figure 3C**).

## 4 Discussion

This study shows that in adults with T1DM, subjective and objectively assessed sleep quality is poor, as occurs in 77% nights analyzed with actigraphy and 52% patients report Pittsburgh index *>* 5. In a previous epidemiological study using the Pittsburgh survey to measure subjective sleep quality (1) in a sample of 222 patients, authors found that 41% have poor sleep quality (Pittsburgh index >5). According to our study’s observational data, sleep quality variability in adults with type 1 diabetes is associated with more significant variability in nocturnal blood glucose levels.

Similar findings have been reported in a group of adolescent patients using actigraphy and CGM, where sleep fragmentation, earlier awakening, and longer duration of WASO are associated with greater glycemic variability and longer time in hypoglycemia (8).

To our best knowledge, this is one of the first studies using machine learning techniques to analyze the relationship between sleep structure and times in normo-, hypo-, and hyperglycemia and to show that better sleep structure is associated with longer time in the glycemic range during that day. Our results confirm those of a previous study in 20 adult patients (38), showing that poor sleep quality is associated with greater glycemic variability. However, they found no association between sleep quality and time in range. This study only analyzes the relationship between sleep quality and glycemia with a linear mixed-effects model. The application of machine learning clustering reveals that nights with a higher disorder of the sleep structure presented lower time in range and a higher percentage of time in hypoglycemia. Increased time in the deep sleep phase was correlated with lower HBA1c and less time in nocturnal hypoglycemia in a previous study (9).

Other previous studies that do not use continuous glucose monitoring also suggest that sleep disturb-ances worsen glucose control. In particular, patients with short sleep duration (<6.5 hours) reported higher HbA1c than patients with longer sleep duration (>6.5 hours) (14). Social jet lag (major changes in the duration and timing of sleep between weekdays and holidays) was associated with worse chronic metabolic control (39). Some studies demonstrate the influence of sleep quality or duration on glycemic control in children. However, the findings among the different authors are not the same: it has been reported that a longer duration of the light sleep phase is associated with higher mean daily blood glucose, more episodes of hyperglycemia, and higher HbA1c (4), and that increased nocturnal awakenings (40) correlate with high glycemic variability.

Most of these studies have limitations in that they were conducted on a small number of patients, some only used subjective sleep assessments, and only two studies in adults simultaneously performed continuous glucose monitoring and polysomnography.

There are several possible mechanisms involved in poorer glycemic control (41). Decreasing the duration of the REM phase would produce lower nocturnal glucose consumption, given that in the cerebral REM phase, glucose consumption is similar to awake. In contrast, in the cerebral non-REM phase, glucose consumption is much lower. In the general population and patients with diabetes, sleep deprivation, fragmentation, and decreased deep sleep are associated with decreased insulin sensitivity, possibly mediated by increased cortisol and Growth hormone (GH) levels. In patients with T1DM, higher nocturnal levels of growth hormone, adrenaline, ACTH, and cortisol than in the control population have been reported (3). In an experimental study, the partial restriction of a single night of sleep (4 hours) de-creased peripheral insulin sensitivity measured by the hyperinsulinemic-euglycemic clamp in patients with T1DM (42).

Although it has been proposed that continuous glucose monitoring may alter sleep quality due to hyper-or hypoglycemia alarms, in this study, the CGM did not have alarms, so the likelihood of inter-ference of the CGM on sleep quality is very low. Finally, sleep disturbances could worsen glycemic control by an indirect mechanism related to patients’ behavior and cognitive functions. An association has been described in children and teenagers between a shorter duration of sleep and a decrease in the frequency of self-monitoring and insulin bolus administration (43).

## 5 Limitations

The use of Fitbit devices is controversial. The previous generation of Fitbit devices was equipped only with body movement sensors and, therefore, were unsuitable for recording sleep stages. How-ever, new generations incorporated heart rate recording and a light sensor, so the Fitbit Ionic models greatly improved the ability to identify sleep stages. In fact, they are considered sleep-staging models (16). However, the data is not available directly from the web. Instead, programming an API is necessary to obtain the data. Although the code was tested thoroughly, a deeper validation with other wrist devices of higher complexity would be beneficial. Recently evaluations of several commercial sleep technologies during sleeping concluded that Fitbit ionic measured with greater accuracy and limited bias Total sleep time (TST), total wake time (TWT), and sleep efficiency (SE). Regarding sleep, stages were reported poor for the time spent in REM sleep and with lower error in the other two stages (44). We did not find other validation studies for Fitbit Ionic. The sample size of n=22 should be considered when evaluat-ing the results and conclusions presented in this work. Although all of the participants are adults and sleep recommendations are not different throughout adulthood, it would be necessary to study better the differences on sleep patterns based on age.

This study did not look at possible differences between patients treated with MDI or CSII. Separating patients by treatment would further reduce the number of nights used to analyze each group as we clustered by night rather than by patient. Future work should include larger samples to investigate such possible relationships.

## 6 Conclusion

To our best knowledge, our work is the first study that, using artificial intelligence and statistical techniques, has found a relationship between sleep structure and times in normo-, hypo-, and hypergly-cemia. Our main conclusion is that better sleep structure is associated with a longer time in the glycemic range. Future studies are needed to confirm these findings in a larger patient population and investigate the mechanisms involved in the decreased time in range and increased glycaemic variability caused by poor sleep quality. We believe that sleep disturbances should be a factor to be assessed in the clinical practice of patients with type 1 diabetes and that strategies should be designed to treat these disturbances. As future work, we are considering conducting a study to investigate further the relationship between sleep and glycemia by age group. Intuitively, variables affected by age, such as habits, responsibilities, social influences, etc., may produce significant differences in sleep patterns and diabetes outcomes.

## Data availability statement

The raw data supporting the conclusions of this article will be made available by the authors, without undue reservation.

## Ethics statement

The studies involving human participants were reviewed and approved by Hospital Universitario Príncipe de Asturias. The patients/participants provided their written informed consent to participate in this study.

## Author contributions

MB-S: Experimental design. Medical analysis. JV: AI techniques, programming, figures, writting. AS-S: statistical analysis OG: AI analysis, writing JIH: AI analysis, writing and Funding. All authors contributed to the article and approved the submitted version.
